# Investigation of the effect of tannic acid on doxorubicin-ınduced testicular damage and functions in a rat model

**DOI:** 10.1007/s00210-025-04238-0

**Published:** 2025-05-10

**Authors:** Emine Toraman, Hamid Ceylan

**Affiliations:** Science Faculty, Department of Molecular Biology and Genetics, Atatürk University, 25240 Erzurum, Turkey

**Keywords:** Doxorubicin, Tannic acid, Oxidative stress, Testicular toxicity, MtDNA copy number

## Abstract

Reproductive and testicular toxicity is one of the most important side effects of doxorubicin (DOX), a chemotherapy drug. In our study, we investigated the ameliorative effect of tannic acid (TA), a polyphenol, against DOX-induced testicular and reproductive toxicity in rats. For this purpose, rats divided into four groups (*n* = 5 each). Rats were treated with DOX cumulatively 30 mg/kg (5 mg/kg by six equal injections) for 2 weeks to induce testicular toxicity. Rats were then treated daily with TA (50 mg/kg) and DOX + TA combination. After treatments, animals were decapitated and testicular tissues were removed. Then, to examine the effect of DOX and TA treatments on oxidative stress, changes in *Sod*, *Cat*, *Gpx*, *Gst*, and *Gr* specific activities and mRNA levels were determined. To determine the effects on inflammation, changes in the expression levels of *Tnf-α*, *IL6*, *Foxo1*, *Foxo3*, *Cox2*, and *Inos* genes were examined. In order to examine the therapeutic effect of TA on the spermatogenesis process, mRNA levels of *Dazl*, *Amh*, and *Ddx4*, which regulate reproductive functions, were determined. Additionally, changes in oxidative damage markers GSH, MDA, 8-OHdG, iNOS, and TNF-a and changes in mitochondrial DNA copy number were also investigated. The results showed that DOX treatment caused a decrease in the levels of oxidative stress and reproductive parameters and an increase in inflammation and DNA damage parameters. However, it was determined that oxidative stress, inflammation, and tissue damage decreased in testicular tissue after TA treatment. In addition, it was observed that TA also improved the expression levels of reproductive genes. When all the data were evaluated together, it was determined that TA administration has a therapeutic effect against the damage and toxicity caused by DOX.

**Supplementary Information:**

The online version contains supplementary material available at 10.1007/s00210-025-04238-0.

## Introduction

Cancer, which occurs when cells multiply uncontrollably, can be treated with methods such as surgery, radiotherapy, and chemotherapy (Valentovic 2018; He et al. 2018). While these methods are efficacious in the treatment of cancer, they are associated with significant adverse effects and damage to multiple organs (Boussada et al. 2017). Testicular toxicity represents one of the most frequently occurring adverse effects associated with this treatment (Sakai et al. 2018). Doxorubicin (DOX) is an anthracycline antibiotic frequently used in tumor treatments (Meredith and Dass 2016). However, its clinical use is limited by its many side effects, including testicular toxicity. Studies have shown that chemotherapeutics can significantly affect spermatogenesis and male fertility (Zavras et al. 2016; Silva et al. 2018; Gurel et al. 2019). It has been demonstrated that oxidative stress and apoptosis in reproductive cells may be responsible for DOX-mediated testicular toxicity. However, the precise molecular mechanisms that lead to testicular damage remain unclear (Coelho et al. 2017; Johnson et al. 2017; Yang et al. 2017; El-Maddawy and Abd El Naby 2019; Mohan et al. 2021).

There are a number of studies suggesting that herbal and bioactive compounds can ameliorate DOX-induced testicular toxicity (Rizk et al. 2014; Kumar et al. 2018; Renu and Valsala Gopalakrishnan 2019). Tannic acid (TA), a natural polyphenol used in many areas such as agriculture and food, has antioxidant and radical scavenging properties and can be used as a food preservative or nutraceutical (Wu et al. 2021; Wang et al. 2022). The available literature on the adverse effects of DOX on the reproductive system is somewhat limited. A review of the literature reveals a need for further comprehensive studies to elucidate the impact of TA on DOX-induced testicular toxicity. Studies have shown that TA has antioxidant and anti-inflammatory properties. However, we did not find any studies on the ameliorative effect of TA in DOX-induced testicular toxicity. Furthermore, the fact that the effect of TA on the regulation of reproductive function and genetic regulation after testicular toxicity will be investigated reveals another unique aspect of our study. In our study, the therapeutic effect of tannic acid, which is a powerful antioxidant and anti-inflammatory agent, against doxorubicin-induced oxidative stress and cellular damage in rat testes was demonstrated. In addition, it was determined that TA can protect reproductive functions by reducing doxorubicin-induced testicular toxicity. There are numerous knowledge gaps that need to be addressed to improve our understanding of the mechanism and mitigation of DOX-induced reproductive toxicity. We aim that this study will contribute to filling this knowledge gap.

## Material and method

### Animals and experimental groups

All experiments and studies were approved by Atatürk University Local Animal Care Commission (Meeting Date: 26.10.2023, Decision No: 180). Experiments were performed on 20 male Sprague Dawley rats weighing 220–295 g obtained from Atatürk University Medical Experimental Research and Application Center (ATADEM). During the experimental period, all rats were housed in transparent cages at 21 ± 2 °C room temperature, 60 ± 10% humidity, 12 h of light, and 12 h of darkness. Ready-to-eat pellet feed and tap water were used to feed the rats during the experiment. It is important to note that no restrictions were imposed on the feed or water consumption of the rats.

Rats were randomly divided into four groups with five animals in each group.

- Group 1 (control): Rats in this group were given saline used as solvent of DOX (Doxorubicin Adriblastina RD) and TA (Sigma/1401–55-4) for 14 days.
- Group 2 (DOX): The induction of DOX toxicity was achieved by the intraperitoneal administration of six equal doses (5 mg/kg) of DOX to rats in this group. To compensate for the stress associated with the injection, all animals were administered normal saline on days when DOX was not administered.
- Group 3 (TA): TA 50 mg/kg was administered intraperitoneally (i.p.) to five animals in this group for 14 days.
- Group 4 (DOX + TA): Rats in this group received 5 mg/kg DOX + 50 mg/kg TA intraperitoneally (i.p.) as described in groups 2 and 3.

The doses used in the study were determined by taking into account similar previous studies (de Veras et al. 2021; Pourmirzaei et al. 2021). After all treatments, all animals were sacrificed under anesthesia with a ketamine/xylazine cocktail, and testicular tissues were collected.

### Determination of glutathione and lipid peroxidation levels

The Sedlak and Lindsay method was employed to measure glutathione (GSH) levels. The supernatant from the homogenized testicular samples was used to measure GSH (mM) with 5,5′-dithiobis-(2-nitrobenzoic acid). The amount of GSH was identified by spectrophotometric absorbance measurement at 412 nm (Sedlak and Lindsay 1968). The thiobarbituric acid (TBA) test was used to determine lipid peroxidation (LPO) levels in rat testis homogenates. MDA reacts with TBA to form a pink-colored product in the said test. The amount of MDA was identified by spectrophotometric absorbance measurement at 532 nm (Ohkawa et al. 1979). The standard curve was obtained using 1,1,1,3,3-tetramethoxypropane (Odabasoglu et al. 2006).

### ELISA analyses

Testis samples were weighed and homogenized in 50 mM Tris–HCl buffer and the supernatant was used to determine the amount of TNF-a, 8-OHdG and iNOS. ELISA test kits, rat tumor necrosis factor α (TNF-α) ELISA Kit (YLA0118RA), rat 8-OHdG ELISA Kit (ELK8994), Reed Biotech Rat NOS2/iNOS (nitric oxide synthase 2, ınducible) ELISA Kit (RE1419R) were used for analysis. 8-OHdG levels were determined using the competitive inhibition enzyme immunoassay technique. Standards or samples are added to the appropriate microtiter plate wells followed by the addition of a biotin-conjugated antibody specific for rat 8-OHdG (1/100 ratio). Horseradish peroxidase (HRP)–conjugated Avidin is then added to each microplate well and incubated. After addition of TMB substrate solution. The enzyme–substrate reaction is terminated by adding sulfuric acid solution and the color change is measured spectrophotometrically at a wavelength of 450 nm. For iNOS measurement, samples are added to micro ELISA plate wells and combined with specific antibody. Then biotinylated detection antibody (1/100) and avidin-horseradish peroxidase (HRP) conjugate are added to each micro-plate well successively and incubated. Substrate solution is added to each well. The enzyme–substrate reaction is terminated by adding stop solution, and optical density (OD) is measured spectrophotometrically at 450 nm wavelength. For tumor necrosis factor α (TNF-α) measurement, samples are loaded into wells. Biotin-labeled anti TNF-α antibodies are then added to combine with streptavidin-HRP forming an immune complex. By adding substrates A and B, the absorbance (OD) of each well is measured individually at 450-nm wavelength.

### Molecular analyses

#### Quantitative gene expression

Testicular samples were taken from the animals in the experimental groups and RNA isolation was performed using an RNA isolation kit (Invitrogen 12,183,025 RNA Mini Kit). From the isolated RNAs, cDNA synthesis was performed using a cDNA synthesis kit (Biolabs, E6300S). Quantitative real-time polymerase chain reaction PCR (qPCR) was used to measure quantitative changes in the expression of *Sod*, *Cat*, *Gpx*, *Gst*, *Gr*, *Tnf- α*, *IL6*, *Foxo1*, *Foxo3*, *Cox2*, *Dazl*, *Ddx4*, and *Amh* genes. PCR reactions were performed on a Rotor-Gene Q (Qiagen-Cat no: 9001560) used to obtain gene expression profiles of cDNA samples. Quantitative gene expression analysis was determined using the SYBR Green (Biorad-Cat no: 10000076382) method (Budak et al. 2014). The PCR reaction mixture was prepared for 25 μl of template DNA, and amplification reactions were carried out as described below: 50 °C for 2 min, 95 °C for 10 min, 45 cycles of 95 °C for 10 s, and annealing/extension at 60 °C for 1 min (Baltacı et al. 2022). Relative gene expression data were analyzed using the ΔCT method (Zhang 2003). The primer sequence information used is given in Table 1.

**Table 1:** Gene-specific primers show the names, gene symbols, and sequences

Gene symbols	Primer	Sequence (5'→ 3')
*Sod1*	F:	GCTTCTGTCGTCTCCTTGCT
	R:	CTCGAAGTGAATGACGCCCT
*Cat*	F:	GCGAATGGAGAGGCAGTGTA
	R:	GTGCAAGTCTTCCTGCCTCT
*GPx*	F:	TGGCTTACATCGCCAAGTC
	R:	CCGGGTAGTTGTTCCTCAGA
*Gst*	F:	AGACGGGAATTTGATGTTTGA
	R:	TGTCAATCAGGGCTCTCTCC
*Gr*	F:	AGTTCACTGCTCCACACATCC
	R:	TCCAGCTGAAAGAACCCATC
*Tnf- α*	F:	AGGAGGGAGAACAGCAACTC
	R:	TGTATGAGAGGGACGGAACC
*IL-6*	F:	AGTTGCCTTCTTGGGACTGA
	R:	TACTGGTCTGTTGTGGGTGG
*Foxo1*	F:	ACCGTATCTGTGTGTGTGTGTG
	R:	ACAGCCAAGTCCATCAAGAC
*Foxo3*	F:	ACTGAGGAAAGGGGAAATGG
	R:	TGCTGGGTTAGGAAGATGGC
*Cox2*	F:	AACACACACAAGCACAATAGACG
	R:	GGAGGGAAGGGCGATTAG
*Dazl*	F:	GTCTTCATCAGCAACCACCAG
	R:	CATCCATCCTAACATCAATTCCAC
*Ddx4*	F:	CAGCATTCCCATTGTGTTAGC
	R:	GGCAGTTATTCCATCCCTCATC
*Amh*	F:	AATACCAGGGGCCTCATCT
	R:	TCAGTACTGCAGACTCCGAA
*Gapdh*	F:	TGGACCTCATGGCCTACATG
	R:	AGGGAGATGCTCAGTGTTGG

### Enzyme activity assays

Testicular samples taken from rats were weighed and homogenized with a phosphate buffer. The supernatant obtained was used for activity measurements. Quantitative protein determination was performed according to the Bradford method (Bradford 1976). The basic principle used in the measurement of superoxide dismutase (SOD) enzyme activity is based on the inhibition of the reduction of nitro blue tetrazolium (NBT) by the SOD enzyme in the homogenate of superoxide radicals produced by an enzymatic reaction in the reaction medium. A 50% inhibition of the reduction rate of NBT during incubation is expressed as one SOD unit (Sun et al. 1988). Catalase enzyme (CAT) activity was measured by modifying the method by Aebi (1984). In the homogenates obtained, catalase activity absorbance was measured at 240 nm wavelength with 15-s intervals for 1 min using a quartz plate (Aebi 1984). In glutathione peroxidase (GPX), activity from the obtained homogenates was determined by adding hydrogen peroxide (H_2_O_2_) at 340 nm and measuring H_2_O_2_ consumption for 5 min (Beutler 1975). GST activity measurement was carried out according to the method of Habig et al. (1974). GST enzyme activity in homogenates was determined by measuring at a wavelength of 340 nm for 3 min (Habig et al. 1974). Glutathione reductase enzyme activity was measured according to the method by Carlberg and Mannervik (1985). Glutathione reductase enzyme activity in homogenates was determined by measuring at a wavelength of 340 nm for 3 min. The enzyme activity (EU/mL) was calculated using the absorbance values obtained. Then, specific activity (EU/mg) was calculated by combining it with the protein determination results (Carlberg and Mannervik 1985).

### Determination of mitochondrial DNA copy number

To determine how TA treatment affected the change in mtDNA copy number after DOX toxicity, mtDNA copy number analysis was performed. Testicular samples were obtained from rats in all experimental groups, and genomic DNA was isolated using a DNA isolation kit (Gene ALL, 108–101). Mitochondrial DNA copy number alterations were analyzed by quantitative polymerase chain reaction (qPCR) using the SYBR Green method. The forward and reverse primer sequences for nuclear gene (18S rRNA) were 5’-GACTCAACACGGGAAACCTC-3’ and 5’-TAACCAGACAAATCGCTCCA-3’, respectively. The forward and reverse primer sequences for mtDNA (D-loop region) were 5’-AGGCATCTGGTTCTTACTTCAG-3’ and 5’-TGACGGCTATGTTGAGGAAG-3’, respectively. The mtDNA copy number was calculated using formula 2 ^(ct mtDNA−nucDNA)^ (Rooney et al. 2015).

### Statistical analysis

All experiments were analyzed using ANOVA, with Tukey's post-hoc comparisons between groups. Statistical analysis of the experimental results was performed using GraphPad Prism software version 8.0 (GraphPad Software, San Diego, CA). All data are presented as Standard Deviation (± SD). A symbol (*) indicates statistically significant changes. *p < 0.05 (significant); * *p < 0301 (very significant); * **p < 03001; and * ** *p < 0.0001 (highly significant). The calculated *p* values are not interpreted as hypothesis tests; they are only descriptive.

## Results and discussion

Found in various plant extracts and foods, TA has antioxidant, antimutagenic, anti-inflammatory, antiviral and anticancer properties (de Veras et al. 2021). The underlying mechanism of DOX-induced testicular injury is characterized by biochemical and physiological tissue disorders resulting from oxidative stress and the generation of reactive oxygen species (ROS) (Sherif et al. 2014; Hassen et al. 2021; Ujah et al. 2021). As a consequence of the search for efficacious anticancer treatments with diminished adverse effects, the development of natural compounds and pharmaceutical agents with reduced cytotoxicity has been initiated (Sarıözkan et al. 2017; Abd-Elrazek et al. 2020; Tharmalingam et al. 2020). This study investigated the potential of TA in the treatment of testicular toxicity and male infertility following chemotherapy exposure.

**Fig. 1:**
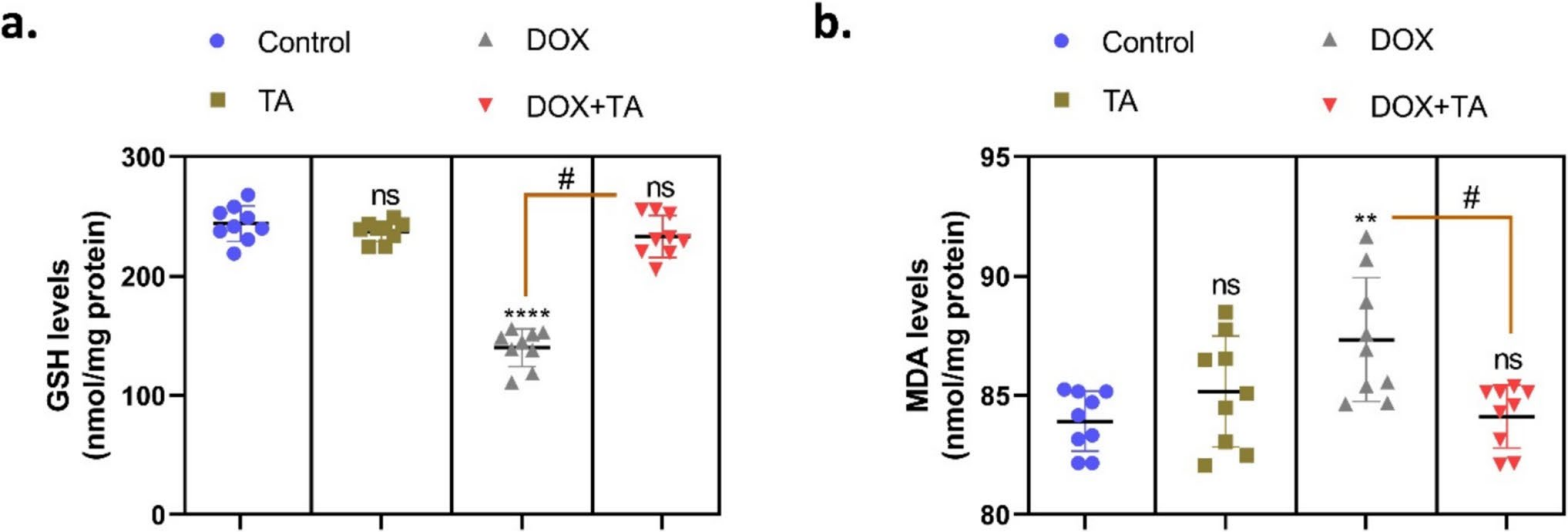
The figure shows the results of measuring glutathione GSH (**a**) and malondialdehyde MDA (**b**) levels in the testicular tissue of rats in all groups. The symbol * indicates the comparison of the groups compared to the control, while the symbol # indicates the degree of statistical significance between the groups

GSH is a compound involved in reducing reactive oxygen species (ROS) and oxidative stress. Decreased GSH levels in cells make them vulnerable to toxic chemicals (Sacks et al. 2018; Lan et al. 2021). In one study, it was determined that GSH levels decreased in rats with testicular damage caused by cisplatin, and the application of arjunolic acid used as a preservative increased the decreased GSH levels (Sherif et al. 2014). ROS, which occurs as a result of decreased GSH levels, causes deterioration in the male reproductive system and sperm function (Kocpinar et al. 2020). Oxidative stress causes lipid oxidation, leading to the production of secondary products such as MDA and exacerbation of oxidative damage (Delkhoshe-Kasmaie et al. 2014). Increased intracellular MDA level is a biomarker for the detection of oxidative damage (Mouro et al. 2019). In another study investigating the effect of curcumin (CUR) on DOX-induced testicular damage in male rats, DOX treatment induced oxidative stress, decreasing sperm motility rate, percentage of viable sperm, cellular antioxidants, and increasing MDA levels. CUR treatment significantly reduced these side effects in a dose-dependent manner compared to the DOX group (Aksu et al. 2019). In a study investigating the effect of melatonin and ozone to eliminate the negative effects of bisulfan, an anticancer agent, on mouse testicular tissue, it was shown that bisulfan caused oxidative damage by increasing MDA levels (Moghadam et al. 2021). In the present study, GSH and MDA levels were measured in the tissues of rats in all groups to investigate the effect of DOX on tissue damage in testicular tissue and the potential of TA to ameliorate this damage. The data obtained showed that GSH levels decreased (*P* < 0.001) (Fig. 1a) and MDA levels increased (Fig. 1b) (*P* < 0.01) in the DOX group compared to the control group. In the DOX + TA group, both GSH and MDA levels normalized and reached a level close to the control group. In this study, the decrease in GSH in testicular tissue after DOX application indicates that antioxidant defense mechanisms are weakened and cells become more vulnerable to free radical damage. The increase in MDA suggests that DOX-induced lipid peroxidation is increased and cellular membranes are subject to oxidative damage. These findings suggest that doxorubicin may impair reproductive functions by increasing oxidative stress and lipid peroxidation in testicular tissue. It was observed that TA administration increased GSH levels, thus strengthening endogenous antioxidant defense, and decreased MDA levels, i.e., suppressing lipid peroxidation and oxidative cell damage. These results suggest that TA may play a protective role against DOX-induced testicular toxicity.

**Fig. 2:**
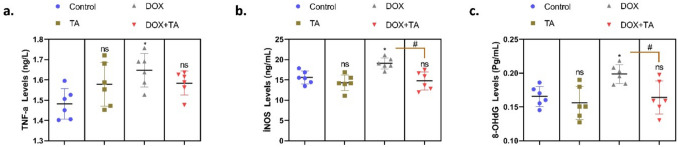
The levels of TNF-a (**a**), İNOS (**b**), and 8-OHdG (**c**) in testicular tissue are presented in the figures. The symbol * indicates the comparison of the groups compared to the control, while the symbol # indicates the degree of statistical significance between the groups

ELISA analyses were performed to investigate the DNA damage and inflammation that DOX application may cause in testicular cells and the therapeutic effect of TA on these damages. 8-OHdG (8-hydroxy-2′-deoxyguanosine), one of the DNA base mutations, is a marker used to detect oxidative DNA damage (Valavanidis et al. 2009; Özcan et al. 2015). Increased levels of inducible nitric oxide synthase (iNOS) also induce oxidative damage by increasing ROS levels in tissues and cells (Rochette et al. 2013). When the data obtained from the study were analyzed, it was found that TNF-a, INOS, and 8-OHdG levels increased significantly in the DOX-treated group compared to the control group (*P* < 0.05) (Fig. 2). After treatment with TA, it was determined that this increase recovered and approached the control. Increased 8-OHdG levels suggest that DOX causes oxidative stress–induced DNA base modifications in testicular cells, which may disrupt cellular genetic stability. Increased TNF-α and iNOS levels suggest that DOX triggers cellular stress mechanisms by increasing the inflammatory response in testicular tissue. Increased iNOS increases nitric oxide (NO) levels, increasing the production of reactive nitrogen species (RNS) and reactive oxygen species (ROS), which may trigger oxidative DNA damage and apoptosis in testicular cells. The reduction of 8-OHdG levels by TA application indicates that DNA base damage is significantly reduced and the genetic stability of testicular cells can be preserved. The reduction of TNF-α and iNOS levels by TA supports the reduction of inflammatory response and alleviation of cellular stress. These findings suggest that TA alleviates DOX-induced testicular toxicity through its strong antioxidant and antiinflammatory properties. The reduction of DNA damage in testicular tissue may contribute to the preservation of the genetic integrity of sperm cells. The reduction of inflammation may support testosterone production and sperm development by reducing the effects of Leydig and Sertoli cell functions. These findings suggest that TA may be considered as a potential adjuvant agent to prevent or attenuate chemotherapy-induced testicular damage.

**Fig. 3:**
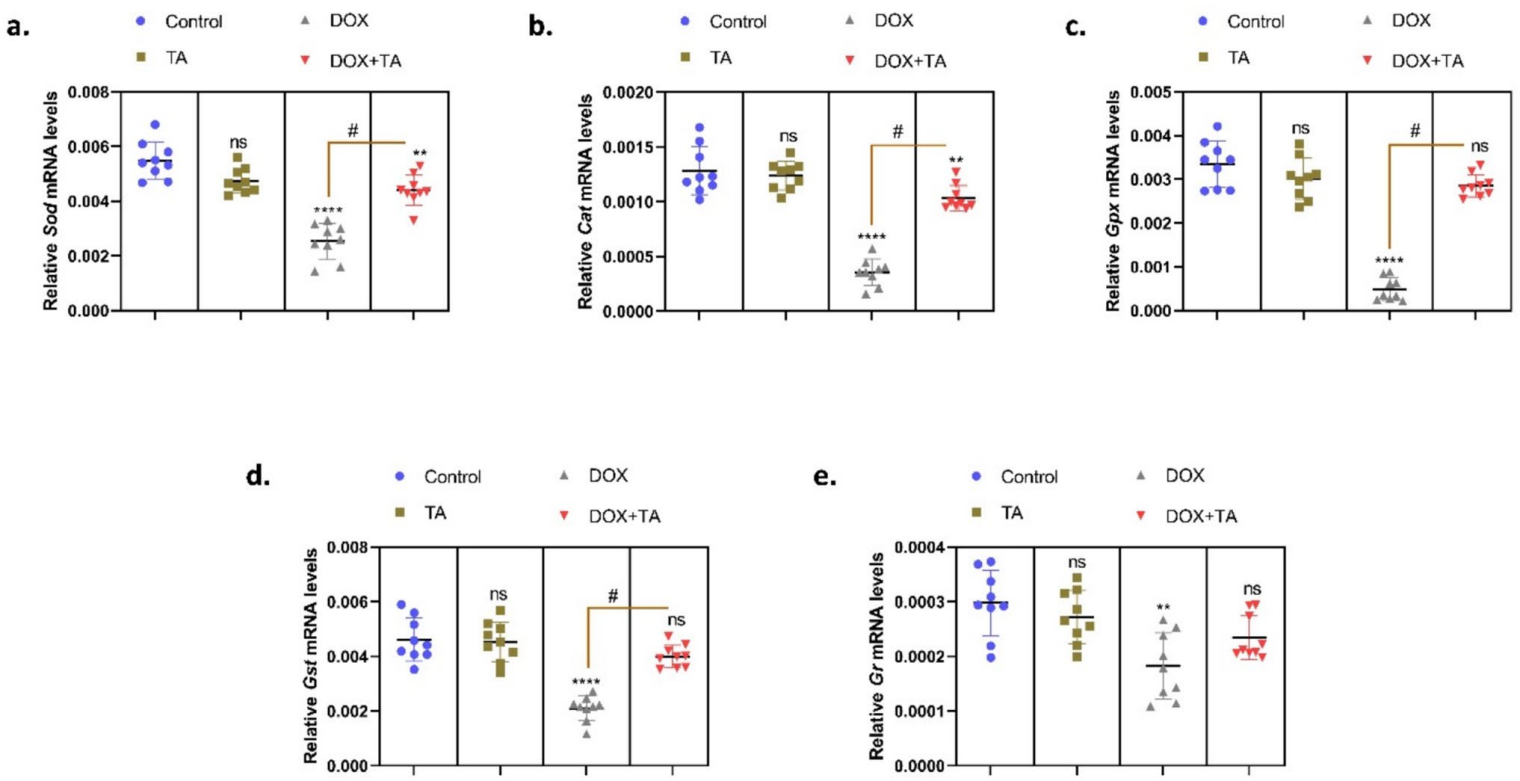
The relative mRNA expression levels of *Sod* (**a**), *Cat* (**b**), *Gpx* (**c**), *Gst* (**d**), and *Gr* (**d**) in testicular tissue are presented in the figures. The symbol * indicates the comparison of the groups compared to the control, while the symbol # indicates the degree of statistical significance between the groups

An increase in ROS levels can cause oxidative stress, which has the potential to damage cells and tissues (Sulukan et al. 2023). The accumulation of ROS that occurs following chemotherapy treatment results in a reduction in antioxidant capacity, which in turn causes testicular damage and disrupts spermatogenesis processes. The most effective method of eliminating the adverse effects of chemotherapy is through antioxidant treatments (Cengiz et al. 2020) (Cadeddu Dessalvi et al. 2021). Oxidative stress, which occurs due to excessive production of ROS and a decrease in antioxidant levels, is prevented by antioxidant defense mechanisms such as SOD, CAT, and GPx in organisms (Sarıözkan et al. 2017). SOD is one of the antioxidant enzymes that sweeps ROS production in cells. It rapidly converts superoxide (O^−2^) anion to low-risk hydrogen peroxide and GPx and catalase to water in cells (Moghadam et al. 2021). In a study, it was observed that in the case of nephrotoxicity induced by cisplatin, an anticancer drug in mice, the level of *Sod*, *Cat*, and *Gpx* gene expressions decreased and caused oxidative stress (El-Beshbishy et al. 2011). In another study investigating the protective effect of astaxanthin obtained from seaweeds on DOX-induced liver damage, it was found that the expression of *Sod*, *Cat*, and *Gpx* genes, whose expression decreased after DOX administration, increased after astaxanthin treatment and reached normal levels (Ma et al. 2020). In our study, in order to clarify the oxidative stress induced by DOX in testicular tissue and the ability of TA to inhibit this oxidative stress, the changes in the expression levels of oxidative stress parameters *Sod*, *Cat*, *Gpx*, *Gst*, and *Gr* genes were examined. When the data were analyzed, it was determined that the expression levels of these genes decreased significantly in the DOX-treated group. This indicates that DOX causes oxidative stress formation and antioxidant system genes are spent to clear this ROS accumulation. In the DOX + TA group, gene expression levels were found to be similar to the control (*P* < 0,001) (Fig. 3). In real-time PCR (qPCR) experiments, the Ct (threshold cycle) values of reference genes such as GAPDH should not be exactly the same between each sample and group, but should be in a similar range. Since the role of these genes is to normalize the expression of target genes, their expression is expected to be stable. However, biological variation, cDNA synthesis efficiency, pipetting errors, RNA quality differences and thermal cycler variations can slightly affect Ct values. Therefore, GAPDH Ct values are not exactly the same in every study. In our study, certain differences were observed in the Ct values of the GAPDH gene between the groups (Supplementary Table 1). Some of these differences may be due to variation between the cDNA samples used. Although the expression levels of reference genes such as GAPDH are ideally expected to be constant, it has been reported in the literature that the Ct range of this gene can vary according to tissue type; for example, GAPDH Ct values in testis tissue can range from 13 to 28 (Zhang et al. 2021; Gao et al. 2024). In this study, all target gene expressions were normalized to GAPDH and calculated by ΔCt method. In addition, for a more holistic evaluation of the data, graphs were prepared using Ct values not normalized by GAPDH and presented in the supplementary material. However, significant differences were observed between normalized and non-normalized data for some genes. For example, a decrease was observed in the normalized data of GPX gene in the DOX group, whereas an increase was observed in the non-normalized data (Supplementary Fig. 1–3). This suggests that variations in GAPDH expression could be a potentially confounding factor in data interpretation. Therefore, we believe that both normalized and non-normalized data should be evaluated together in the interpretation of the results obtained. The variability of GAPDH in some groups may create uncertainty, especially in the interpretation of low-level gene expression changes. Considering this uncertainty, the data in our study are presented in both ways in order to present the data transparently to the reader and to clearly indicate these limitations. Considering this uncertainty, we performed analyses based on both GAPDH-normalized (ΔCt method) and raw Ct values in order to present the data transparently to the reader and to clearly indicate these limitations. However, in direct comparisons with Ct values, it is known that gene expression decreases as Ct value increases. Therefore, for some genes, the non-normalized Ct plots show an opposite orientation from the normalized log2(fold change) plots. This contrast is due to the fact that increasing Ct value implies decreasing expression. Thus, direct comparison of raw Ct values may be limited in reflecting the true biological variation of gene expression, especially due to the observed between-group variation in GAPDH expression. This may introduce contrasts in the interpretation of non-normalized data.

**Fig. 4:**
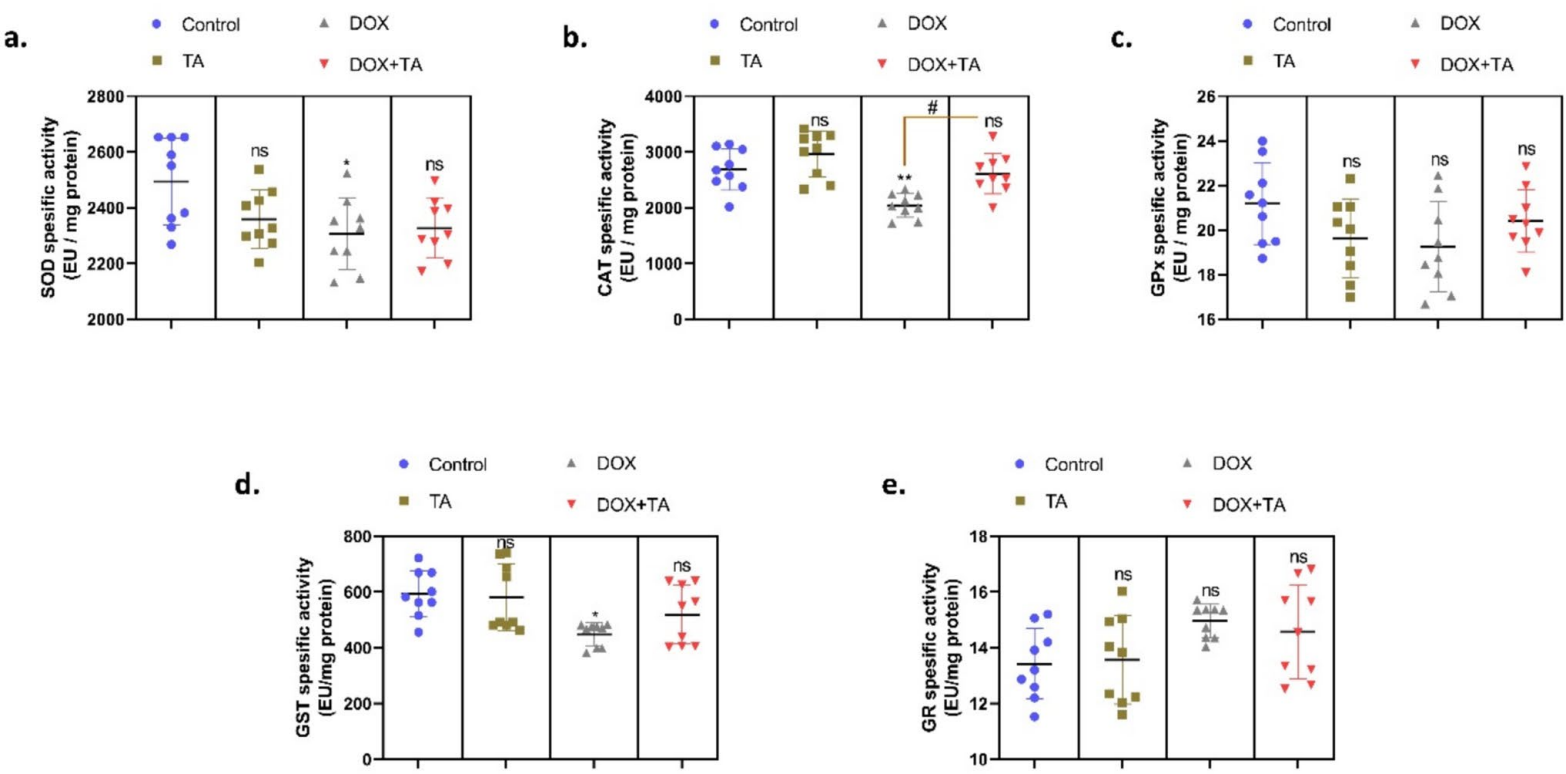
The figure shows the specific activities of SOD (**a**), CAT (**b**), GPx (**c**), GST (**d**), and Gr (**e**) enzymes in rat testicular tissues in all groups. The symbol * indicates the comparison of the groups compared to the control, while the symbol # indicates the degree of statistical significance between the groups

Similar results were observed when enzyme activity analysis data were evaluated (*P* < 0.05) (Fig. 4). The above-mentioned results indicate that TA exhibits a therapeutic effect in attenuating DOX-induced testicular toxicity and stimulates the enzymatic antioxidant system at gene and enzymatic levels. Considering the results, it seems that the accumulation of ROS caused by DOX may lead to a decrease in the activity and gene expression levels of antioxidant enzymes, leading to increased oxidative stress in the testicular tissue. This situation reveals that cellular defense mechanisms have difficulty tolerating the oxidative load caused by DOX. In our study, the determination of *Sod*, *Cat*, *Gpx*, *Gst*, and *Gr* gene expression levels in the DOX + TA group was close to the control group, indicating that TA prevents the antioxidant system deterioration caused by oxidative stress and returns gene expression levels to normal. These results indicate that TA can activate cellular defense systems that fight oxidative stress and thus protect testicular tissue from oxidative damage.

**Fig. 5:**
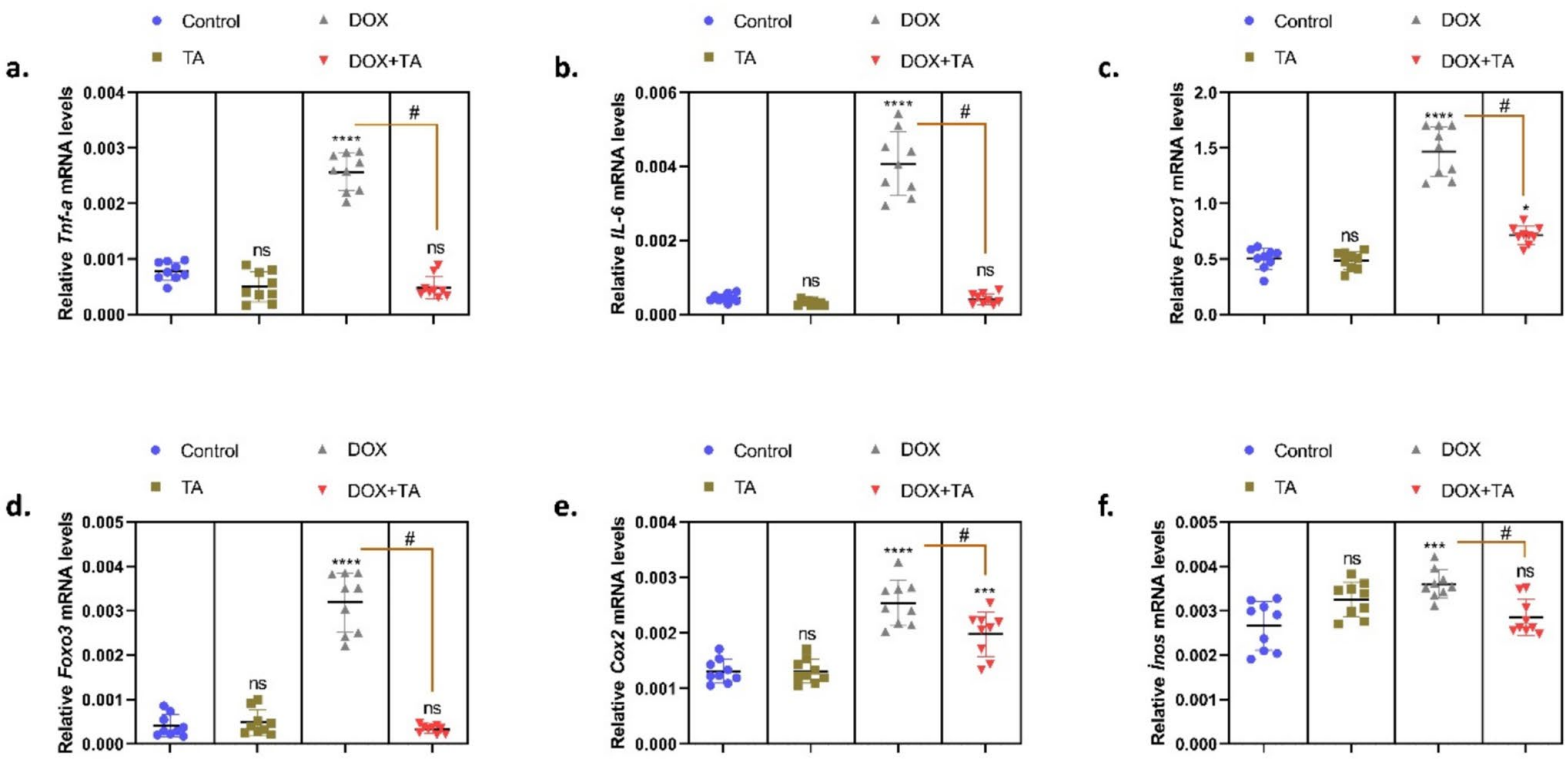
The relative mRNA expression levels of *Tnf-a* (**a**), *IL-6* (**b**), *Foxo1* (**c**), *Foxo3* (**d**), *Cox2* (**e**), and *Inos* (**f**) in testicular tissue are presented in the figures. The symbol * indicates the comparison of the groups compared to the control, while the symbol # indicates the degree of statistical significance between the groups

Recurrent inflammatory damage has long been thought to play a role in all stages of cancer. It seems that cancer drugs, including DOX, can cause inflammation in different organs, including the testis (Cha et al. 2018). Several studies have shown that the levels of proinflammatory cytokines increased significantly following DOX administration (Fouad et al. 2019; Huyut et al. 2021; Ujah et al. 2021). In another study, there was a clear link between oxidative stress and the inflammatory response, including cytokine release, after DOX treatment (Bien et al. 2007). The data demonstrated that DOX-induced an imbalance in immune regulation, resulting in a notable elevation in the expression levels of pro-inflammatory genes, including *Tnf-a*, *IL-6*, *Foxo1*, *Foxo3*, *Cox2*, and *Inos* (*P* < 0.0001) (Fig. 5), in comparison to the control group. The increase in genes such as *Tnf-a*, *IL-6*, *Cox-2*, *İnos*, *Foxo1*, and *Foxo3* indicates that DOX triggers inflammation and activates cellular death mechanisms. The reversal of these processes by TA treatment suggests that it suppresses the inflammatory response and restores immune homeostasis in the testicular tissue. This anti-inflammatory effect of TA suggests that it can be evaluated as a therapeutic agent against chemotherapy-induced testicular toxicity.

**Fig. 6:**
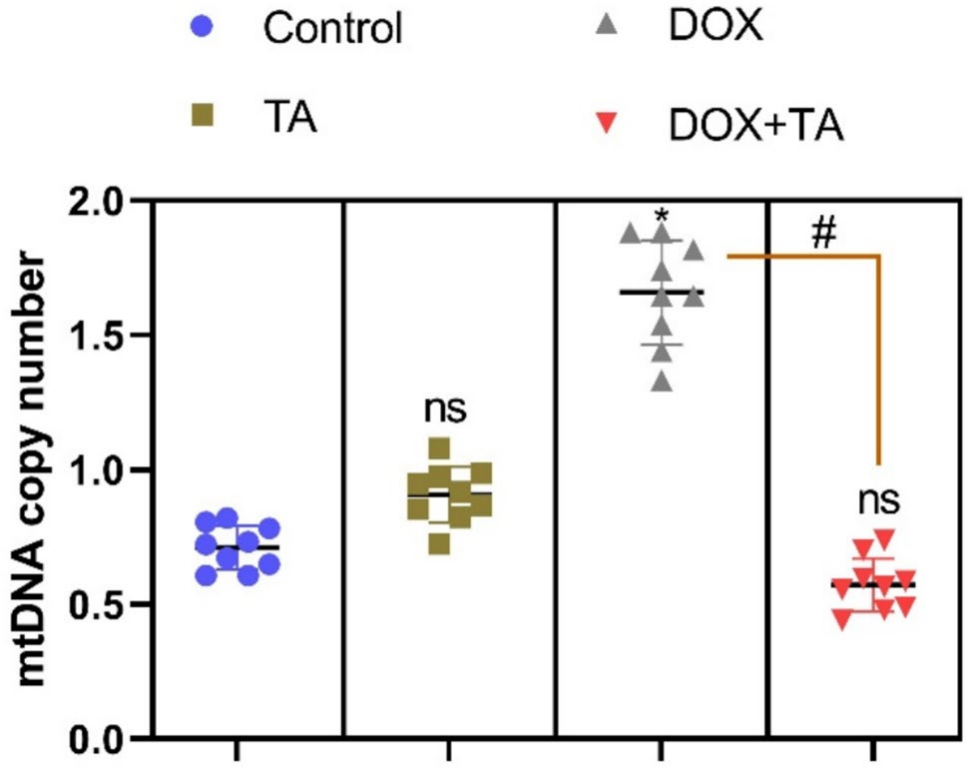
The figure shows the mitochondrial DNA (mtDNA) copy number expressed in testicular tissues of rats in all groups. The symbol * indicates the comparison of the groups compared to the control, while the symbol # indicates the degree of statistical significance between the groups

When there is a functional disorder in the mitochondria organelle, which is responsible for regulating cellular metabolism, a decrease in cellular energy production occurs and a corresponding increase in (ROS) levels occurs (Guyatt et al. 2018). Mitochondrial DNA copy number (mtDNA-CN), a measure of mitochondrial DNA (mtDNA) levels in cells, is not a definitive measure of mitochondrial function, but studies have shown that mtDNA-CN changes are caused by ROS formation, oxidative damage, and inflammation (Castellani et al. 2020; Mori et al. 2022). In our study, mtDNA copy number increased in the DOX-treated group compared to the control group, whereas it decreased in the DOX + TA group and reached the same level as the control group (*P* < 0.001) (Fig. 6). This revealed that oxidative stress and inflammation induced by DOX also caused significant changes in mtDNA copy number. However, TA treatment showed a protective and ameliorative function against the negative effects of DOX. These results suggest that TA may be evaluated as a mitochondrial protective agent against chemotherapy-induced testicular toxicity and may play a potential role in future clinical applications.

**Fig. 7:**
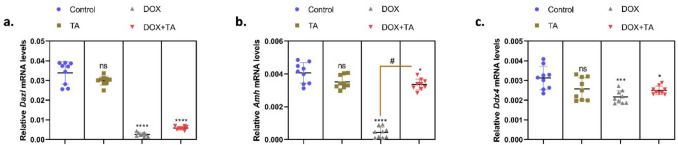
The relative mRNA expression levels of *Dazl* (**a**), *Amh* (**b**), and *Ddx4* (**c**) in testicular tissue are presented in the figures. The symbol * indicates the comparison of the groups compared to the control, while the symbol # indicates the degree of statistical significance between the groups

The Dazl (DAZ-like) gene is a gene responsible for the development and differentiation of embryonic germ cells in vertebrates. Genetic loss or mutation of this gene has been associated with infertility in both vertebrates and invertebrates (Tung et al. 2006; Rosario et al. 2016). The DEAD-box helicase 4 (*Ddx4*) gene, an RNA helicase expressed in germ cells, is closely associated with the development of the reproductive system. Studies have shown that defects in the *Ddx4* gene cause infertility and abnormalities in reproductive functions (Azizi et al. 2021; Amirian et al. 2022). Anti-Müllerian hormone (AMH) is a hormone involved in male sex differentiation and the regulation of reproduction. Abnormalities in *Amh* gene functions can cause dysplasia in the gonads. A study has shown that AMH protein is highly expressed during the non-reproductive period of seasonal reproduction in animals and may be responsible for restricting reproductive activities (Xu et al. 2019; An et al. 2023). In a similar study, it was determined that paclitaxel may cause disorders in reproductive functions by decreasing the expression levels of *Dazl*, *Ddx4*, and *Amh* genes in rat testicular tissue. Parthenolide, a plant component, was found to be able to bring the expression levels of these genes to normal levels (Toraman et al. 2024). In our current study, it was determined that DOX application significantly reduced the expression levels of *Dazl*, *Ddx4*, and *Amh* genes, which are genes that control reproductive functions (*P* < 0.001) (Fig. 7). When we look at the group treated with DOX and TA, it was determined that there was an increase in the mRNA levels of Amh and *Ddx4* genes, and they reached similar levels to the control. This decrease in *Dazl* expression may reduce sperm production by inhibiting the survival and maturation of spermatogonial stem cells. The lack of increase in *Dazl* expression after TA treatment may suggest that TA does not have a direct regulatory effect on *Dazl*. However, the suppression of oxidative stress and inflammation by TA may have indirectly prevented further suppression of *Dazl*. These results suggest that DOX causes impairments in spermatogenesis via *Dazl* pathways and that TA may attenuate the negative effects, although it cannot completely reverse this process. The decrease in *Ddx4* expression may lead to loss of spermatogonial stem cells and impaired sperm production. TA treatment restored *Ddx4* expression levels to values close to the control group. This suggests that TA may contribute to the protection of stem cells that play a critical role in spermatogenesis. It may be thought that the antioxidant and anti-inflammatory effects of TA increase *Ddx4* expression by protecting spermatogonial cells from oxidative damage. These findings suggest that TA may reverse the harmful effects of DOX by protecting germ cell health via *Ddx4* pathways. The increase in *Amh* expression levels after TA treatment suggests that TA helps Sertoli cells maintain their functions by protecting them against oxidative and inflammatory damage.

In this study, the effects of DOX on testicular toxicity and the protective potential of TA were investigated at the level of basic molecular pathways. For the evaluation of reproductive functions, gene expressions related to spermatogenesis, cellular stress responses, inflammatory processes, oxidative stress markers, and mitochondrial dynamics were analyzed. In future studies, sperm parameters, hormonal analyses, and fertility tests are recommended to confirm the effects of TA on reproductive functions. However, the current findings provide strong evidence that TA may have a protective effect on testicular cells at the molecular level.

## Conclusion

DOX, a chemotherapy drug, has been shown to have toxic effects on reproductive organs, especially on the testes. However, despite extensive research, the exact mechanism by which DOX exerts its harmful effects on the male reproductive system remains unclear (Gurel et al. 2019). Plant-derived compounds may be effective agents in the treatment of oxidative stress caused by chemotherapy drugs (Sznarkowska et al. 2017; Asadi-Samani et al. 2019). Data from this study suggest that TA can reduce DOX-mediated oxidative stress and damage in testicular tissue, reduce inflammation, and improve reproductive toxicity. The data will help us understand the fertility problems that cancer patients may face after chemotherapy and provide a better understanding of how to develop treatments to help them maintain their fertility.

## Supplementary Information

Below is the link to the electronic supplementary material.

Supplementary file1 (DOCX 460 kb)

## Data Availability

All source data for this work (or generated in this study) are available upon reasonable request.
