# Supplementary material for: Investigation of the effect of tannic acid on doxorubicin-ınduced testicular damage and functions in a rat model
Source: Naunyn Schmiedebergs Arch Pharmacol. 2025 May 10;398(11):15587–98. doi: 10.1007/s00210-025-04238-0 (PMC12552281; doi:10.1007/s00210-025-04238-0)
Supplement: Supplementary file 1 — Supplementary file1 (DOCX 460 kb) [file 210_2025_4238_MOESM1_ESM.docx]

**Table 1.** CT values ​​of GAPDH gene in each group

| **Control** | **TA** | **DOX** | **DOX+TA** |
| --- | --- | --- | --- |
| 22,13 | 20,38 | 15,22 | 18,86 |
| 21,69 | 20,42 | 16,7 | 18,5 |
| 22,05 | 20,21 | 16,32 | 19,9 |
| 21,63 | 19,32 | 15,73 | 19,41 |
| 21,35 | 19,7 | 16,8 | 19,42 |
| 21,37 | 20,23 | 16,47 | 19,65 |
| 22,02 | 19,73 | 15,99 | 19,64 |
| 21,58 | 19,21 | 16,25 | 19,34 |
| 21,01 | 19,28 | 15,78 | 19,27 |

*The mean (± SD) Ct values ​​for each group were 21.65 +/- 0.37, 19.82 +/- 0.49, 16.14 +/- 0.51 and 19.33 +/- 0.43, respectively.


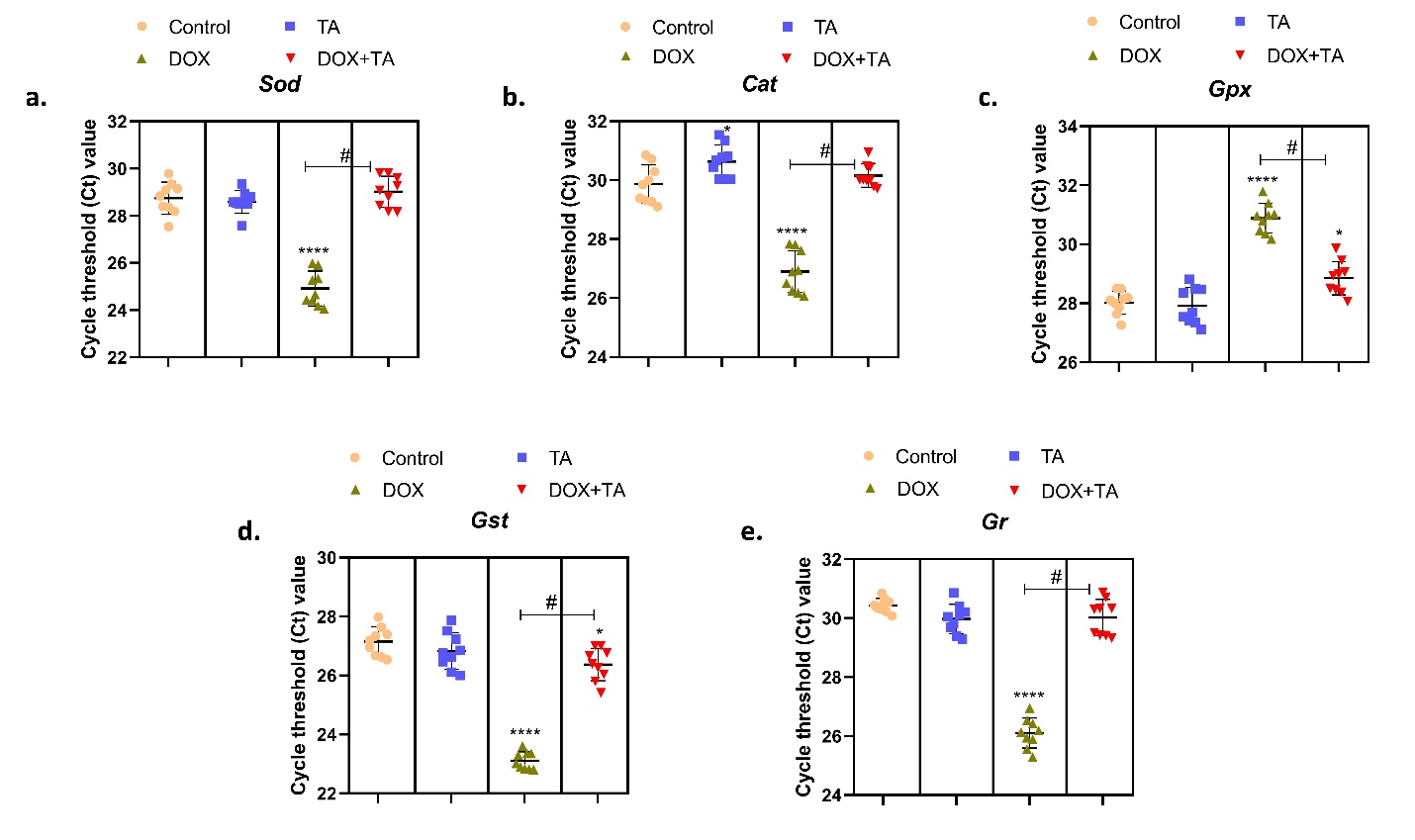


**Figure 1.** Graphs drawn using CT values ​​of target genes *Sod* **(a),** *Cat* **(b),** *Gpx* **(c),** *Gst* **(d)** and *Gr* **(d)** and changes between groups. The * symbol shows the comparison of groups according to the control, while the # symbol shows the degree of statistical significance between groups.


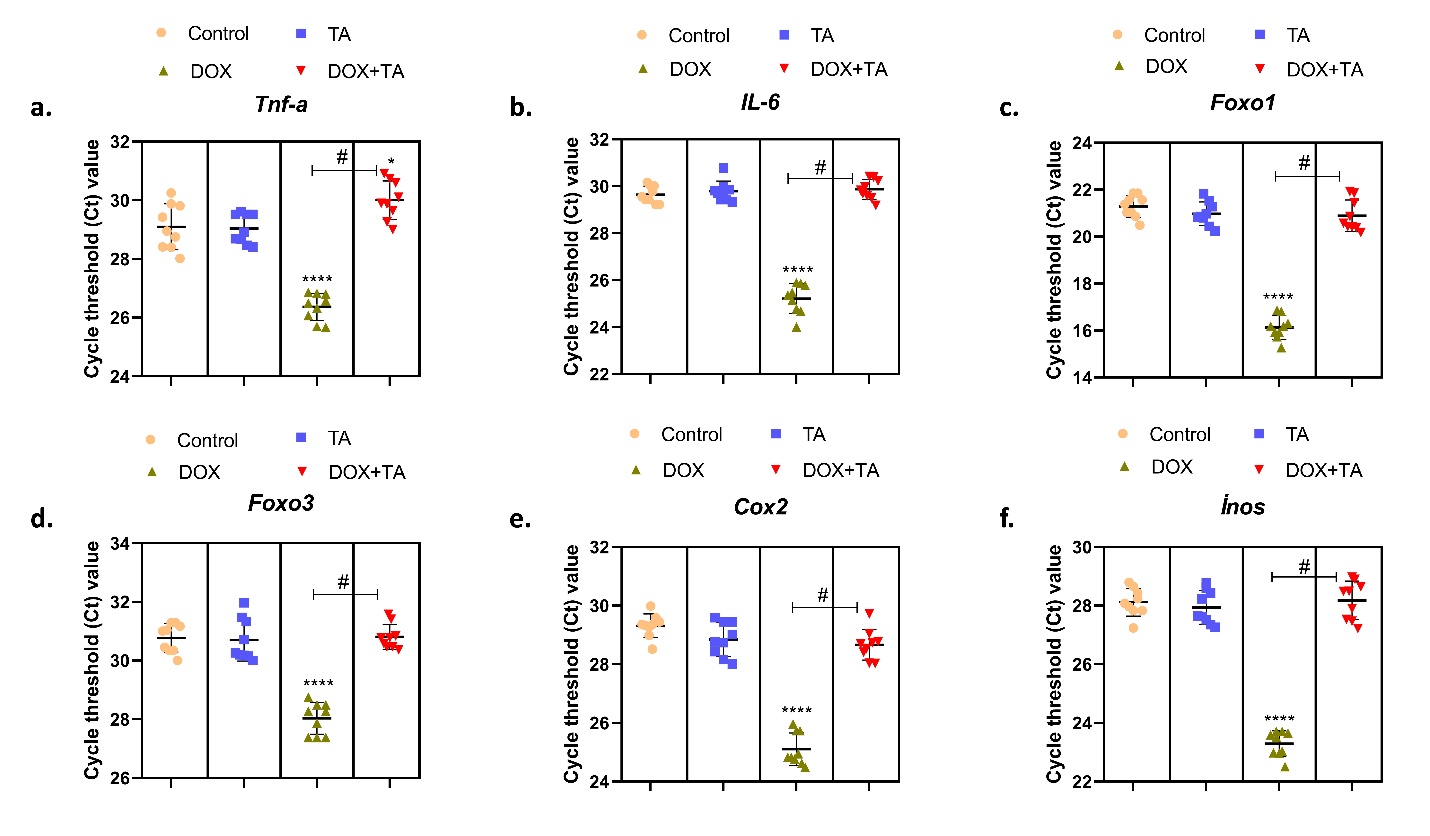


**Figure 2.** Graphs drawn using CT values ​​of target genes *Tnf-a* **(a)**, *IL-6* **(b)**, *Foxo1* **(c)**, *Foxo3* **(d)**, *Cox2* **(e)**, and *Inos* **(f)** and changes between groups. The * symbol shows the comparison of groups according to the control, while the # symbol shows the degree of statistical significance between groups.


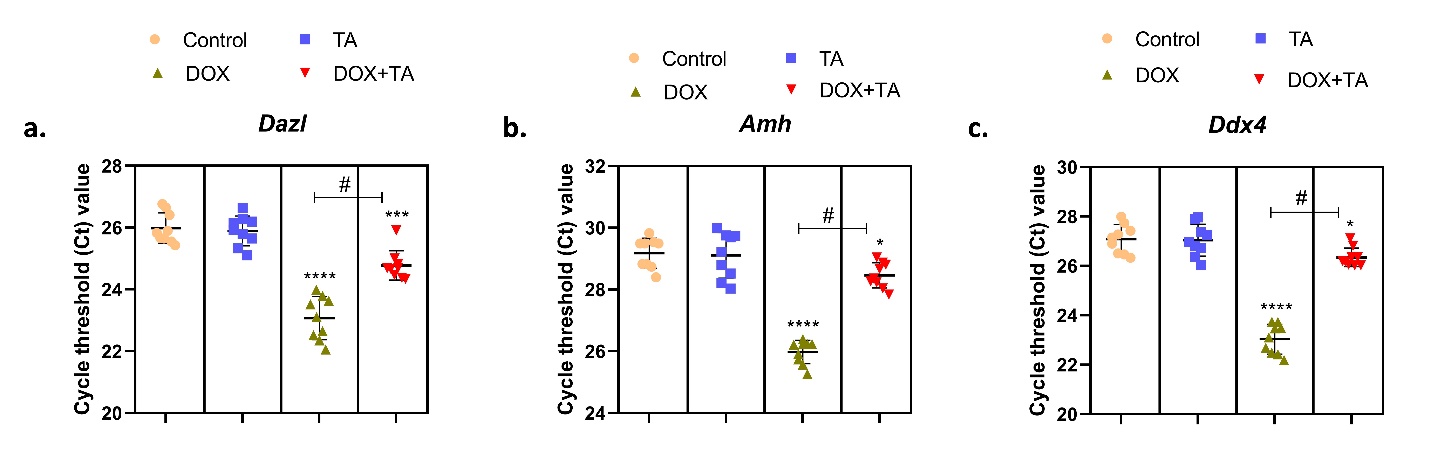


**Figure 3.** Graphs drawn using CT values ​​of target genes *Dazl* **(a)**, *Amh* **(b)**, and *Ddx4* **(c)** and changes between groups. The * symbol shows the comparison of groups according to the control, while the # symbol shows the degree of statistical significance between groups.
